# Application of Artificial Intelligence in Cardio-Oncology Imaging for Cancer Therapy–Related Cardiovascular Toxicity: Systematic Review

**DOI:** 10.2196/63964

**Published:** 2025-05-09

**Authors:** Hayat Mushcab, Mohammed Al Ramis, Abdulrahman AlRujaib, Rawan Eskandarani, Tamara Sunbul, Anwar AlOtaibi, Mohammed Obaidan, Reman Al Harbi, Duaa Aljabri

**Affiliations:** 1 Research Office, Johns Hopkins Aramco Healthcare, Medical Access Road 1, Dhahran, Saudi Arabia, 966 556373411; 2 College of Medicine, University College Dublin, Dublin, Ireland; 3 College of Medicine, Royal College of Surgeons in Ireland-Bahrain, Muharraq, Bahrain; 4 Emergency Department, King Fahad Medical City, Riyadh, Saudi Arabia; 5 Health Informatics Department, Johns Hopkins Aramco Healthcare, Dhahran, Saudi Arabia; 6 College of Medicine, University of Jeddah, Jeddah, Saudi Arabia; 7 College of Public Health, Imam Abdulrahman bin Faisal University, Dammam, Saudi Arabia

**Keywords:** artificial intelligence, cardiology, oncology, cancer therapy–induced, cardiotoxicity, cardiovascular toxicity, machine learning, imaging, radiology

## Abstract

**Background:**

Artificial intelligence (AI) is a revolutionary tool yet to be fully integrated into several health care sectors, including medical imaging. AI can transform how medical imaging is conducted and interpreted, especially in cardio-oncology.

**Objective:**

This study aims to systematically review the available literature on the use of AI in cardio-oncology imaging to predict cardiotoxicity and describe the possible improvement of different imaging modalities that can be achieved if AI is successfully deployed to routine practice.

**Methods:**

We conducted a database search in PubMed, Ovid MEDLINE, Cochrane Library, CINAHL, and Google Scholar from inception to 2023 using the AI research assistant tool (Elicit) to search for original studies reporting AI outcomes in adult patients diagnosed with any cancer and undergoing cardiotoxicity assessment. Outcomes included incidence of cardiotoxicity, left ventricular ejection fraction, risk factors associated with cardiotoxicity, heart failure, myocardial dysfunction, signs of cancer therapy–related cardiovascular toxicity, echocardiography, and cardiac magnetic resonance imaging. Descriptive information about each study was recorded, including imaging technique, AI model, outcomes, and limitations.

**Results:**

The systematic search resulted in 7 studies conducted between 2018 and 2023, which are included in this review. Most of these studies were conducted in the United States (71%), included patients with breast cancer (86%), and used magnetic resonance imaging as the imaging modality (57%). The quality assessment of the studies had an average of 86% compliance in all of the tool’s sections. In conclusion, this systematic review demonstrates the potential of AI to enhance cardio-oncology imaging for predicting cardiotoxicity in patients with cancer.

**Conclusions:**

Our findings suggest that AI can enhance the accuracy and efficiency of cardiotoxicity assessments. However, further research through larger, multicenter trials is needed to validate these applications and refine AI technologies for routine use, paving the way for improved patient outcomes in cancer survivors at risk of cardiotoxicity.

## Introduction

The World Cancer Research Fund International reported 18.1 million cancer cases in the year 2020, with breast and lung cancer being at the top of the list, representing 12.5% and 12.2% of all cases, respectively [1]. Breast cancer is the most commonly diagnosed type of cancer globally [2]. In 2020, the International Agency for Research on Cancer reported 27,885 new cancer cases, with nearly 47% of these cases ending with death [3].

In the United States, there are currently 17 million cancer survivors; by 2030, that number is predicted to rise to 22 million. For many cancer survivors, cardiovascular disease (CVD) is the leading cause of noncancer morbidity and mortality. Studies show that compared to the general population, patients with cancer have a 2‐6 times higher chance of dying from CVD. Considering the progress made in cancer therapies and the decrease in cancer-related fatalities, comprehensive cardiovascular care is essential to improving these patients’ overall results [4].

In recent years, there has been a notable advancement in the fight against cancer. However, a new problem has come to light: the potential for lifesaving cancer treatments to cause unintended damage to the heart. This is where cardio-oncology, a rapidly developing field, comes into play. It focuses on the crucial relationship between cancer treatment and heart health, focusing on controlling and preventing cardiovascular toxicity [5].

Cardiovascular toxicity, commonly known as cardiotoxicity, defined by the 2022 European Society of Cardiology Cardio-Oncology guidelines, is the term used to describe the harm inflicted upon the heart muscle or cardiovascular system due to different cancer treatments. Although chemotherapy and radiation therapy are essential tools in the fight against cancer, they can have negative side effects on the heart. These adverse effects can include anything from mild alterations in cardiac function to potentially fatal issues, including heart failure [6].

According to the American Society of Clinical Oncology (ASCO), the survivorship of cancer in the United States is approximately 67% and 18% for 5 and 20 years or more after diagnosis, respectively [7], especially if diagnosed early [8]. However, patients receiving cancer treatments such as chemotherapy, radiotherapy, and targeted agents have a 20% chance of developing myocardial dysfunction, with up to 7% to 10% having cardiomyopathy or heart failure [9]—in other words, therapy-induced cardiotoxicity [10,11]. Therapy-induced cardiotoxicity depends on the type of treatment, such as mediastinal and left-sided radiotherapy, anthracycline-based chemotherapy, and trastuzumab (targeted therapy), and other risk factors such as age, stage of diagnosis, ethnicity, and pre-existing CVDs [12].

Trastuzumab is a targeted therapy that uses drugs and other substances to precisely identify and attack specific types of cancer cells [13]. It is a humanized immunoglobulin G1 monoclonal antibody that is used to treat HER2+ (human epidermal growth factor receptor 2) breast cancer. Recently, it has also been approved to treat HER2+ advanced gastric cancer. The use of trastuzumab on patients with HER2+ breast cancer, which constitutes 20% of breast cancer cases, has demonstrated a significant reduction in recurrence risk, morbidity, and mortality. However, not all patients with HER2+ breast cancer respond to trastuzumab treatment due to resistance [14]. Recently, targeted therapy has been increasingly used in treating cancer, which has resulted in a significant improvement in the overall survival of patients with cancer. However, it can cause systemic toxicity, particularly cardiovascular toxicity [15].

Moreover, one of the most effective chemotherapy agents for several cancer types is anthracycline-based chemotherapy [16]. The American National Cancer Institute defines anthracycline as a type of antibiotic extracted from certain types of *Streptomyces* bacteria; it kills cancer cells by causing damage to their DNA and interfering with their reproduction [17,18]. The anthracycline chemotherapy agents include doxorubicin, epirubicin, daunorubicin, idarubicin, mitoxantrone, and valrubicin [18]. Although anthracyclines have been proven effective in treating various types of cancer, they do not come without adverse effects, which can limit their therapeutic potential [16]. These adverse effects range from mild and short-term to severe and long-term side effects [19]. Thus, early detection of cardiac dysfunction or cardiotoxicity allows the administration of the appropriate cardiac care, improving the overall outcome [20].

Long-term, dose-dependent risks of cardiotoxicity with anthracyclines are well-established [19]. Therefore, the recommended current practice by ASCO is a comprehensive assessment before initiating the treatment that includes a history and physical examination, screening for CVD risk factors, and an echocardiogram [21]. ASCO also recommends that clinicians manage modifiable cardiovascular risk factors (smoking, hypertension, diabetes, and obesity); the clinicians may incorporate several strategies, such as the use of dexrazoxane for cardioprotection, continuous infusion, or liposomal formulation of doxorubicin during the administration of anthracycline therapy [21]. In addition to cardiac imaging during the routine clinical assessment before therapy initiation (echocardiogram and cardiac magnetic resonance imaging [MRI]), ASCO recommends routine surveillance for cardiac function in patients considered to be at increased risk of developing cardiac dysfunction or heart failure [21,22].

The current method for cardiac function surveillance is “echocardiography” [14] to assess the left ventricular ejection fraction (LVEF) and the global longitudinal strain (GLS) [23]. Echocardiography has many advantages, making it the first modality of choice to monitor cardiotoxicity. These advantages include its ability to provide real-time imaging; availability and accessibility; noninvasiveness; and low cost [23]. However, echocardiography has limitations that hinder the detection of early signs of cardiotoxicity. Some of these limitations include the fact that echocardiography is entirely user-dependent, subjectivity in results interpretation, and variability in the image quality [23]. These limitations can result in the inability to detect subclinical cardiotoxicity and the early signs of cardiac dysfunction, which are crucial for personalized treatment plans that aim to improve the patient’s prognosis [23]. Moreover, other CVD manifestations, such as myocardial perfusion and mitochondrial dysfunction, may precede a myocardial injury detected by echocardiography; this can only be recognized by a higher level of imaging modalities, which use targeted radiotracers such as cardiac magnetic resonance imaging (CMR) and nuclear imaging to provide information on specific mechanisms of cardiotoxicity [24].

With the recent emergence of artificial intelligence (AI) and machine learning (ML), their applications have meritoriously contributed to many advancements, with a promising potential for more across different areas, including imaging in the medical field [23,25]. One of the potential advancements is the rise of stable diffusion, a generative model; it is anticipated that it might fill the gap in low-quality medical images by generating data on the missing details of the pathology with pattern recognition [25,26]. AI can generate this data by processing large amounts of readily available imaging data through artificial neural networks inspired by the connectionism of the biological neural network in the brain [25]. Tasks executed by AI algorithms in medical image processing include image acquisition, analysis, segmentation, feature extraction, visualization, registration, and classification [25]. Using AI-augmented imaging in the assessment of cardiotoxicity can help in recognizing subclinical cardiotoxicity caused by anthracyclines in addition to being able to reproduce the images more accurately by enhancing the imaging quality produced by the echocardiograph, which eventually will allow better monitoring and earlier detection of cardiac dysfunction [27]. For more detailed definitions of cancer treatments, cardiovascular toxicity, imaging modalities, and the application of AI in healthcare, please refer to Multimedia Appendix 1.

Despite its potential, the evidence base of AI imaging solutions for cardiovascular care in general and predicting cardiotoxicity in particular has been limited to date. Therefore, further research about AI’s usefulness and effectiveness in the routine practice of cardio-oncology care is necessary. This systematic review aims to review the available literature on the use of AI in cardio-oncology imaging to predict cardiotoxicity and describe the possible improvement of each modality for cardio-oncology imaging when deploying AI to routine practice.

## Methods

This systematic review was conducted according to the PRISMA (Preferred Reporting Items for Reviews and Meta-Analyses) guidelines from July 1 to August 1, 2023. The review is registered in PROSPERO (International Prospective Register of Systematic Reviews; CRD42023446135).

### Search Strategy

The literature search for this review was performed using PubMed, MEDLINE, Cochrane Library, CINAHL, and Google Scholar for relevant studies from inception until June 2023. An AI research assistant (Elicit) was also used to search for relevant papers using the same terminology. In addition, PROSPERO was searched for ongoing similar systematic reviews. The first and senior authors are experienced in systematically reviewing the literature and have published several reviews. In addition, the authors have consulted experts using Editage services to achieve a high level of reliability. Please see Multimedia Appendix 2 for a detailed search strategy.

### Terminology

In order to achieve the objective of this review, the databases were searched using keywords and their Medical Subject Headings (MeSH) terms connected by the Boolean operators “AND,” “OR,” and “*.”

The search used the following terms and their MeSH terms: artificial intelligence, AI, deep learning, machine learning, cardio-oncology, cardiotoxicity, cardiac toxicity, cancer treatment, cancer therapy, “artificial intelligence,” “machine learning,” “AI augmentation,” “deep learning,” OR “AI” AND “Cardio-oncology,” “Cardiotoxicity,” “Cardiovascular toxicity,” OR “Cardiac toxicity” AND “chemotherapy,” “anthracycline,” “cytotoxic regimens,” “immunotherapy,” “Cancer treatment,” OR “therapy-induced” AND “Imaging,” “Echocardiogram,” “Echo*,“ “Cardiac magnetic resonance imaging,” “CMR,” “Multigated acquisition,” “MUGA,” “Cardiac computed tomography,” OR “CCT.”

### Eligibility Criteria and Study Selection

Original studies reporting AI outcomes in adult patients diagnosed with any type of cancer and undergoing cardiotoxicity assessment were included. Outcomes included incidence of cardiotoxicity, LVEF, risk factors associated with cardiotoxicity, heart failure, myocardial dysfunction, signs of cancer therapy–related cardiovascular toxicity (CTR-CVT), echocardiography, and CMR. Non-English studies, case reports, literature reviews, studies on children, and studies that did not include CTR-CVT were excluded from this review.

### Quality Assessment

The first, second, and third authors (MR, HM, and AR) independently assessed the included articles according to the 42-item Checklist for Artificial Intelligence in Medical Imaging (CLAIM) [28]. CLAIM is modeled after the Standards for Reporting of Diagnostic Accuracy Studies guideline. It addresses the application of AI in medical imaging, including classification, image reconstruction, text analysis, and workflow optimization [28]. Subsequently, the first, second, and third authors cross-checked each other’s articles, and conflicts were resolved through group discussion.

### Risk of Bias

Finally, both HM and AO independently assessed the risk of bias for each study across ROBINS-I’s (risk of bias in nonrandomized studies - of interventions) 7 domains: confounding, selection of participants, classification of exposures, deviation from intended exposure, missing data, measurement of outcomes, and selection of reported results. Each domain was rated as low, moderate, serious, or critical based on the each domain’s algorithm, with the most severe rating across all domains determining the overall assessment for each study. Any disagreements in the assessments were discussed until a consensus was reached, with one reviewer (DJ) ensuring consistent application of judgments. Additionally, we engaged a fifth-year medical student experienced in systematic reviews and various research projects to independently evaluate the risk of bias for all included studies using the same tool.

### Statistical Extraction and Analysis

According to the CLAIM checklist, the first, second, and third authors (MR, HM, and AR) extracted data from the included studies. All discrepancies were resolved after a discussion, with HM acting as an arbitrator. Descriptive information about each study was recorded, including publication details (author, year, and country), sample size, cancer type, imaging technique, AI model, outcomes, and limitations. AO performed analysis, and figures were generated using RStudio (version 2023.06.0; Posit PBC).

## Results

### Study Selection

**Figure 1.**
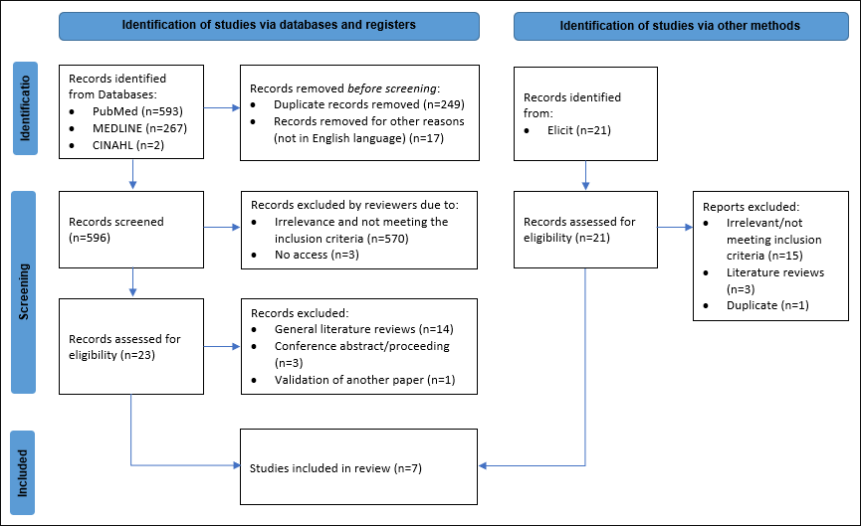
PRISMA flow diagram of the systematic search of the databases for artificial intelligence in cardio-oncology imaging. PRISMA: Preferred Reporting Items for Reviews and Meta-Analyses.

A total of 883 articles were identified in the database search, comprising 593 articles from PubMed, 267 from MEDLINE, 2 from CINAHL, and 21 from Elicit. After eliminating duplicate titles and articles in non-English languages, 617 articles remained. Then, the title and abstract of the 617 articles were screened independently by the first and second authors (MR and HM), and 44 remained. The authors reviewed full texts and 7 articles met the inclusion criteria (Figure 1).

### Quality Assessment of Included Studies

**Figure 2.**
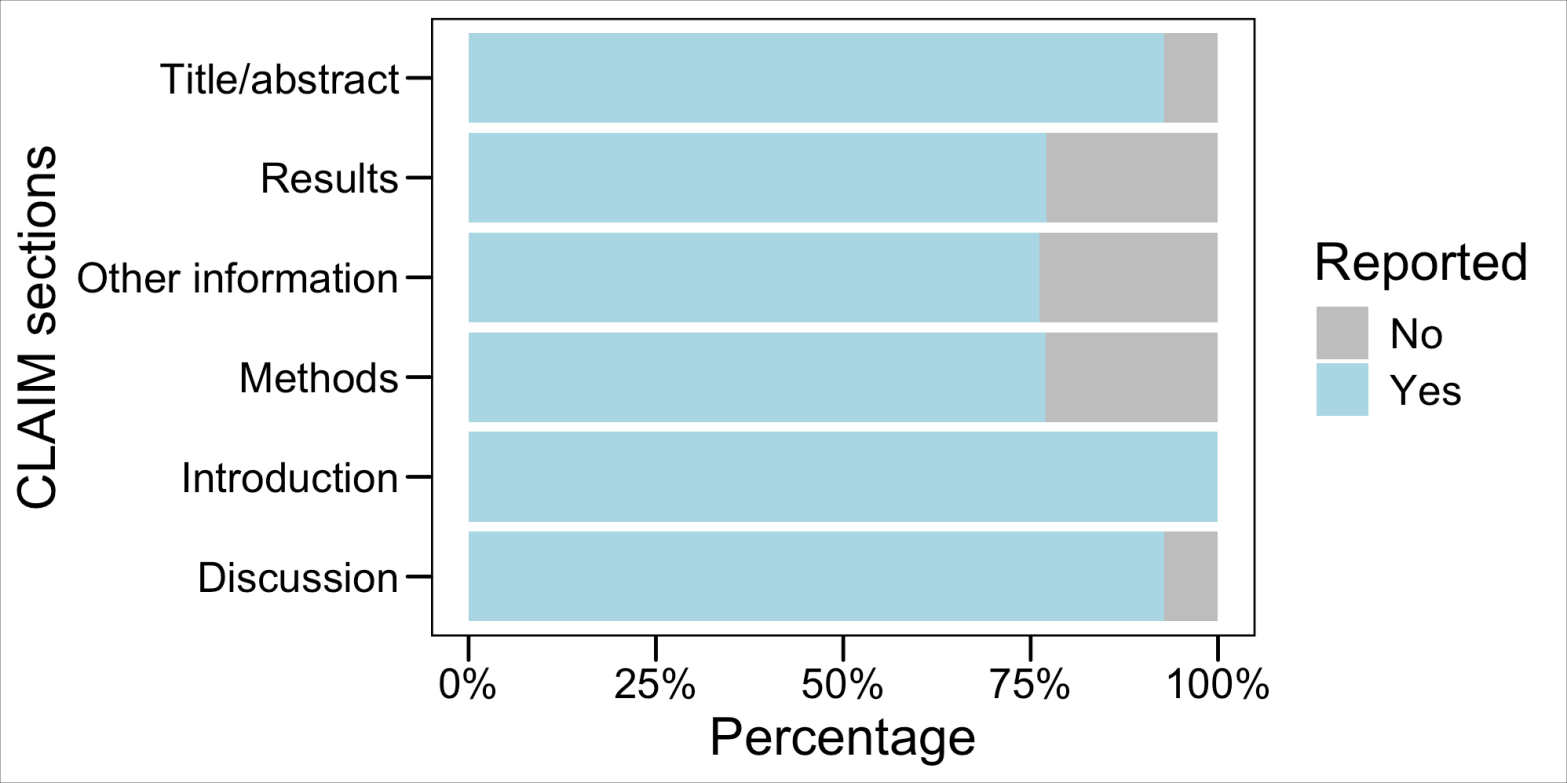
CLAIM sections compliance. CLAIM: Checklist for Artificial Intelligence in Medical Imaging.

The quality assessment used the 42-item CLAIM. The distribution and percentages of different sections and items of CLAIM compliance are depicted in Figures2  3. These sections include title/abstract, introduction, methods, results, discussion, and other information. Each section is categorized into “No” and “Yes” groups, indicating whether it is reported in the selected articles.

**Figure 3.**
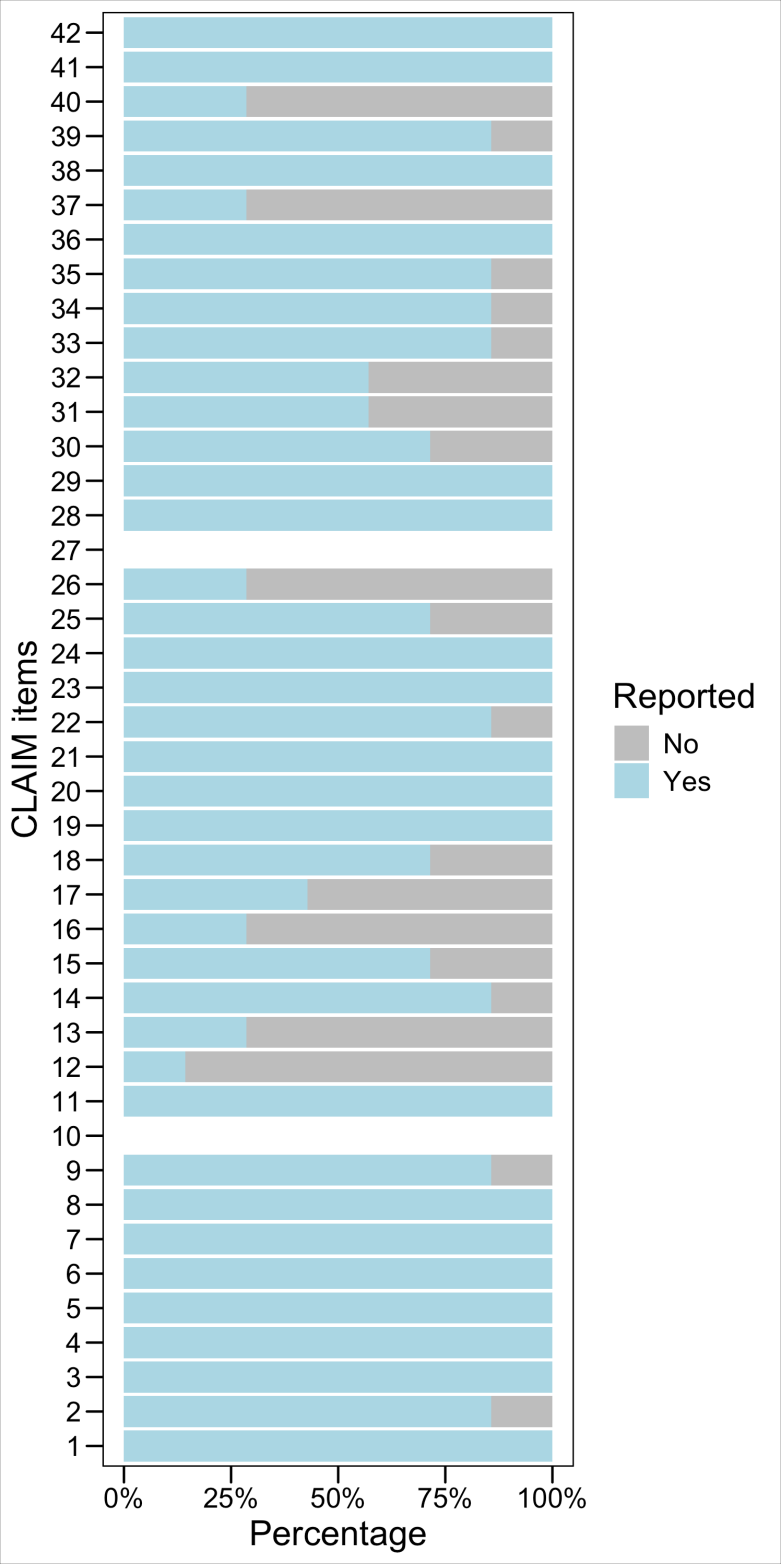
CLAIM items compliance. CLAIM: Checklist for Artificial Intelligence in Medical Imaging.

Based on the data, the title/abstract section was compliant in 93% of the articles (1 article was compliant with the title but not the abstract, which was considered as half compliant). An introduction section was included in all 7 articles, representing 100% compliance. Methods had 77% compliance, results represented 77% compliance, and there was a discussion in 93% of the articles, while other information was 76% compliant. Items 5 and 6 of the checklist—specific to the study methodology and design—were met as follows: 5 studies were conducted prospectively, while the remaining 2 were conducted retrospectively. Moreover, 4 studies were reported as feasibility studies, 2 were exploratory studies, and 1 was a model creation study. Finally, items 10 and 27 of the CLAIM criteria did not apply to the 7 studies.

### Characteristics of the Included Studies

In total, 7 studies conducted between 2018 and 2023 were included, with 5 from the United States (Kar et al [29-31], Zhang et al [32], Edalati et al [33]), 1 from China (Shen et al [34]), and 1 from Taiwan (Chang et al [35]). Of these, 6 studies involved patients with breast cancer with additional cancers (eg, sarcoma, lymphoma, leukemia) in some cohorts. Imaging modalities included MRI (4 studies: 3 displacement encoding with stimulated echoes [DENSE] MRI, 1 CMR), echocardiography (n=2), and nongated, noncontrast chest computed tomography (CT) (n=1). AI approaches varied: 4 studies (57%) used convolutional neural networks (CNNs), 1 (14%) used ML, and 6 (86%) implemented image segmentation. Table 1 provides demographic and descriptive data and Table 2 provides details of the AI components of the included studies.

**Table 1.** Summary of the studies included in this review: demographic and descriptive data.

Author, year, country	Study design	Sample size	Gender	Age (years)	Treatment	Cancer type	Imaging technique
Shen et al, 2023, China [34]	Retrospective, multicenter	N=1468	Male: n=785, female: n=683	>60: n=617, <60: n=851	Anthracycline	Diffuse large B-cell lymphoma	Nongated and noncontrast chest computed tomography for coronary artery calcium scoring echocardiography for cancer therapy–related cardiac dysfunction and major adverse cardiovascular event
Chang et al, 2022, Taiwan [35]	Prospective, single center, with 3 years of follow-up	N=211	n=211	55.8 (SD 10.28)	Anthracycline, trastuzumab	Breast cancer; stage I: n=50; stage II: n=101; stage III: n=52; stage IV: n=8	Echocardiography
Kar et al, 2023, United States [31]	Prospective	N=32	Female n=32	Baseline: 59.4 (SD 9.7); 3 months: 59.6 (SD 9.7); 6 months: 59.6 (SD 9.7)	Anthracycline, trastuzumab, radiotherapy	Breast cancer	DENSE^a^ Magnetic resonance imaging Transesophageal echocardiogram
Kar et al, 2022, United States [30]	Prospective	IG^b^: n=30; CG^c^: n=30	IG female: n=30; CG female: n=30	IG: 54 (SD 9), CG: 50 (SD 13)	Anthracycline, trastuzumab	Breast cancer	DENSE Magnetic resonance imaging
Kar et al, 2021, United States [29]	Prospective	N=42	Female: n=42	55.5 (SD 8.6)	Anthracycline, trastuzumab	Breast cancer	DENSE Magnetic resonance imaging
Zhang et al, 2018, United States [32]	Retrospective, 10 years	Hypertrophy cardiomyopathy: n=260; echo: n=14,035;amyloidosis: n=81; CIC^d^: n=152; pulmonary arterial hypertension: n=27	CIC female: n=152	CIC: 55	Trastuzumab, pertuzumab	Breast cancer	Echocardiography
Edalati et al, 2022, United States [33]	Prospective	CG: n=10, IG: n=10	CG male: n=5; CG female: n=5: IG male: n=5; IG female: n=5	CG: 52.6 (SD 21.2); IG: 47.6 (SD 13.6)	Not applicable	Breast cancer: n=4, sarcoma: n=3, lymphoma: n=1, leukemia: n=1, myeloma: n*=*1	Cardiac magnetic resonance imaging

a DENSE: displacement encoding with stimulated echoes.

b IG: intervention group.

c CG: control group.

d CIC: chemotherapy-induced cardiotoxicity.

**Table 2.** Summary of the studies included in this review: details of the artificial intelligence components in the included studies.

Author, year, country	Artificial intelligence solution	Main outcomes	Limitation
Shen et al,2023, China [34]	Artificial intelligence coronary artery calcium scoring: Deep learning algorithm Image segmentation Bound the range of the heart area Detect and segment the calcified lesions in coronary arteries Calculate coronary artery calcium score	Cancer therapy–related cardiac dysfunction Major adverse cardiovascular events	A larger sample is needed to validate the model’s accuracy The study was limited to Chinese patients
Chang et al, 2022, Taiwan [35]	Machine learning: Multilayer perceptron A tree-based estimator was used to compute essential features, and 15 features were included in our multilayer perceptron model based on experts’ judgments.	Cancer therapy–related cardiac dysfunction Symptomatic heart failure with reduced ejection fraction	A relatively small number of included patients
Kar et al, 2023, United States [31]	Validated advanced artificial intelligence methodologies (DeepLabV3+) with fully convolutional networks: Segmenting the DENSE^a^ magnitude images for chamber quantification Segmenting the DENSE phase images for phase-unwrapping and 3D strain analysis	Global longitudinal strain Cancer therapy–related cardiac dysfunction Adverse cardiac events	Single-center study without external validation No integration between cancer therapy–related cardiac dysfunction risk analysis by combining circulating troponin levels with global longitudinal strain measurements for a practical bivariable prognostic approach
Kar et al,2022,United States [30]	An FCN^b^-based solution adapted from the DeepLabV3+ network: Phase-unwrapping FCN. Compared with conventional unwrapping techniques, validation via phantom setup with known displacements and 3D strain analysis in healthy patients. Left ventricular volume was estimated with previously validated DeepLabV3+. Computation of 3D myocardial strains with the meshfree Radial Point Interpolation Method	Global longitudinal strain	Comparing the performance of phase unwrapping with DeepLabV3+ to another FCN such as PhaseNet. The relationship between the wrapped phase and wrap count can be leveraged with more arbitrary shapes rather than round and ellipsoidal shapes only.
Kar et al, 2021, United States [29]	An automated left ventricular chamber quantification tool (deep learning): DCNN^c^ and DeepLabV3+ with ResNet-50 backbone Some layers of the original ResNet-50 to tailor DCNN for cardiac image segmentation DENSE-based results were validated by corresponding steady-state free precession data in the same patients who were trained using an identical DeepLabV3+ DCNN. Chamber quantification and strain analysis were done after the image-based reconstruction of the full 3D left ventricle.	Left ventricular end diastolic diameter Left ventricular ejection fraction Myocardial strains analyzed with the radial point interpolation method	Backbone networks such as Xception, Inception, ResNet-101, U Net, and others were not tested for left ventricular segmentation.
Zhang et al, 2018, United States [32]	A computer vision pipeline for automated 2D echocardiogram interpretation: Convolutional neural network for view classification Image segmentation Measurements of cardiac structure and function disease detection	Automated identification of 23 viewpoints segmentation of cardiac chambers across 5 common views Quantification of structure and function Detection of hypertrophic cardiomyopathy Detection of cardiac amyloid Detection of pulmonary arterial hypertension	Problems with segmentation Forced normalization to the lower strain value because of the lack of electrocardiogram information, which can result in biases in measurements, estimate of strain Lack of distinguished diagnosis of hypertrophy cardiomyopathy, amyloid, or any hypertrophic disease Lack of comparison of deep learning models to onesbuilt using hand-selected features (left atrial mass or septal thickness)
Edalati et al, 2022, United States [33]	EasyScan: Otsu method: segment heart region Trained regression network: distance map calculation	Scan time difference Accuracy of cardiac plane prescriptions Signal to noise ratio Contrast to noise ratio Overall image quality (sharpness and magnetic resonance image degradation) Ejection fraction Absolute wall thickening	N/A^d^

a DENSE: displacement encoding with stimulated echoes.

b FCN: fully convolutional network.

c DCNN: deep convolutional neural network.

d N/A: not applicable.

The included studies revealed significant clinical heterogeneity across the studies. Study designs ranged from retrospective (eg, Shen et al [34]: n=1468; Zhang et al [32]: n=260) to prospective (eg, Chang et al [35]: n=211; Kar et al [29-31]: n=32‐42), impacting sample size and follow-up duration (eg, 3 years in Chang et al [35] vs 10 years in Zhang et al [32]). Imaging modalities differed in application: echocardiography (Chang et al [35], Zhang et al [32]) assessed LVEF and GLS; DENSE MRI (Kar et al [29-31]) focused on strain analysis; CT (Shen et al [34]) targeted coronary artery calcium scoring (CACS); and CMR (Edalati et al [33]) evaluated image quality and efficiency. AI techniques showed varied sophistication—CNNs (eg, DeepLabV3+ in Kar et al [29-31], CNN pipeline in Zhang et al [32]) and deep learning (Shen et al [34]) enhanced segmentation and classification, while ML with multilayer perceptron (Chang et al [35]) predicted outcomes like heart failure with reduced ejection fraction.

Outcomes centered on CTR-CVT, with cancer therapy–related cardiac dysfunction assessed in 5 studies (Shen et al [34], Chang et al [35], Kar et al [31], Zhang et al [32], Edalati et al [33]), GLS in 3 (Kar et al [29-31]), and LVEF in 3 (Kar et al [29], Edalati et al [33], Zhang et al [32]). Shen et al [34] uniquely linked CACS to major adverse cardiovascular events (MACE). At the same time, Edalati et al [33] emphasized scan time and signal-to-noise ratio. AI improved detection accuracy (eg, automated CACS in Shen et al [34], GLS computation in Kar et al [30,31]) and efficiency (eg, EasyScan in Edalati et al [33]) compared to manual methods. However, direct comparisons across studies were limited by outcome diversity.

Common limitations included small sample sizes (eg, Chang et al [35], Edalati et al [33]), single-center designs (eg, Kar et al [31], Chang et al [35]), and lack of external validation (eg, Kar et al [31]). Geographic restriction (Shen et al [34], Chinese patients) and technical challenges (eg, segmentation issues in Zhang et al [32]) further constrained generalizability.

### Risk of Bias Assessment

**Figure 4.**
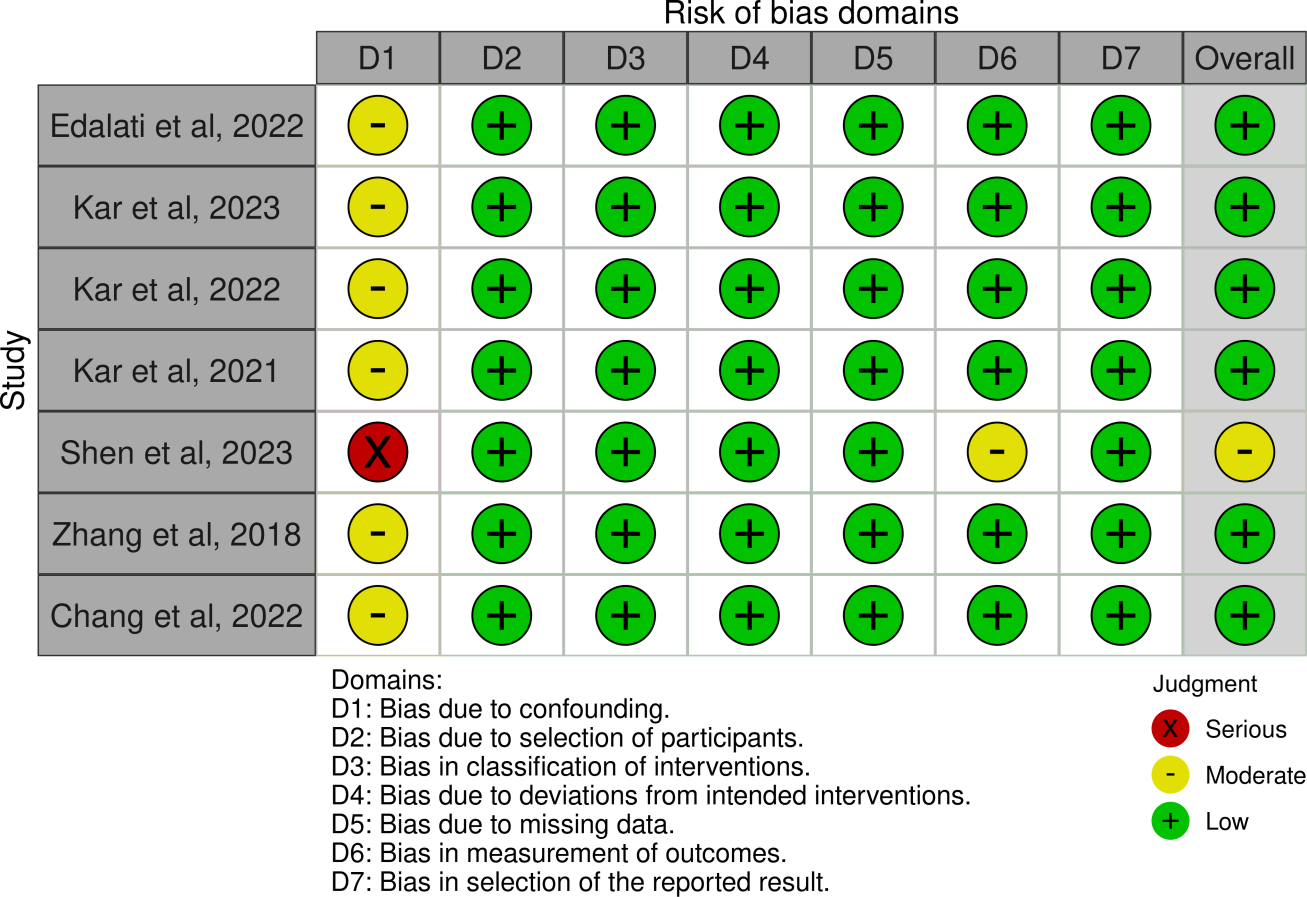
Traffic light plot of risk of bias assessment.

The risk of bias assessment began with general considerations for all studies, which included establishing a minimal set of confounders identified by the reviewers as likely to introduce bias in the observed associations. Next, each study was described individually within the framework of an ideal target trial. The consensus results from the evaluations of the 7 nonrandomized studies are depicted in the “traffic light” plot shown in Figure 4.

## Discussion

### Summary of Included Studies

In 2018, Zhang et al [32] published their work on automating echocardiographic cardiac images using 14,035 echocardiograms collected retrospectively spanning 10 years. Their study included 152 patients diagnosed with CTR-CVT and other patients with other heart conditions such as hypertrophic cardiomyopathy (n=260), amyloidosis (n=81), and pulmonary arterial hypertension [32]. Zhang developed a model for view classification in just a few steps. First, they taught the machine to recognize individual echocardiographic views, where models were trained using manual labels assigned to individual images. Then, they used deep learning architecture for view classification, designed to mimic how the visual system works [32]. This process refers to multiple layers of neurons, processing nodes tuned to recognize features within an image. Afterward, they trained a 13-layer CNN and assessed the accuracy using 5-fold cross-validation. Finally, they used t-distributed stochastic neighbor embedding (an algorithm for visualizing high-dimensional data) to cluster the output of the top layer to visualize the output of their view classification network [32]. By training the CNNs, Zhang could perform image segmentation to locate cardiac chambers that derived cardiac structure and function measurements to develop disease classification models [32]. Zhang’s approach is intended to enable data mining and knowledge extraction from the enormous number of archived echocardiograms, which will have a significant clinical impact by introducing relatively low-cost quantitative metrics into clinical practice and enabling causal insights that require systematic longitudinal tracking of patients [32]. The study results favored using AI-automated measurements over manual measurements across 11 internal consistency metrics. One of these is the correlation between left atrial and left ventricular volumes. This work is argued to have laid the basis for using automated interpretation to support serial patient tracking. Limitations to the study are the length of the analysis period and room for bias. Moreover, the study did not include the number of males or females involved, which may affect the results.

Using a different imaging modality, Edalati et al [33] developed EasyScan, which is automated cardiac planning, by developing, training, and validating 2 deep neural networks on preacquired cardiac MRI datasets (also known as cardiovascular magnetic resonance). EasyScan is implemented with the CMR scanner for automatic slice planning and shimming. The trial included 10 healthy individuals (5 males and 5 females) and 10 cardio-oncology patients (5 males and 5 females) undergoing 2 identical CMR protocols (manual cardiac planning versus AI-based EasyScan) to assess the time difference and accuracy of the cardiac plane. Moreover, Cine images were obtained for the study participants with standard cardiac volume shim and AI-shim to assess the signal-to-noise ratio, contrast-to-noise ratio, overall IQ (sharpness and magnetic resonance image degradation), LVEF, and absolute wall thickening [33]. EasyScan demonstrated accelerated cardiac exams compared to standard manual cardiac planning and achieved an improved and more uniform B0 magnetic field homogeneity using the AI-shim technique compared to volume shimming [33]. Eldalati argued that his results suggest many potential positive outcomes of implementing AI, including a more straightforward and faster workflow chain by minimizing technique complexity. However, a significant limitation of this study is the cohort size, as it is considered small compared to other papers in this field.

Kar et al [29-31] used AI, deep learning, segmentation, and fully convolutional networks (FCN) on the DENSE MRI sequence imaging modality in their 3 studies. In the study published in 2021, Kar et al [29] investigated the automation of measuring left-ventricular strain with a quantification tool via segmentation with a supervised deep convolutional neural network (DCNN) before strain analysis with DENSE images [29]. Kar and her team were able to introduce a novel and automated DCNN architecture–based chamber quantification methodology for detecting the extent of left-ventricular myocardium in single-scan DENSE MRI for patients with breast cancer susceptible to cardiotoxicity. Kar et al identified accurate segmentation, chamber quantification, and subsequent strain analysis in the myocardium as the main critical requirements for engineering and developing this solution. After validation, Kar et al emphasized that their DCNN-based segmentation can provide accurate estimates of the left-ventricular chamber quantification required in strain analysis.

Kar et al argued that their model can perform fast and inexpensive automated measurements of cardiac strain as the model can detect altered material properties. However, the thresholds that define cardiac dysfunction caused by cancer therapy are still an area that needs to be further studied [29].

In 2022, Kar and her team continued their work using DENSE in developing another direct MRI-based, FCN-based, deep-learning semantic segmentation approach for computing GLS for patients with breast cancer [30]. This time, they computed myocardial strains directly from the unwrapped phases with the radial point interpolation method. They compared the results of 30 patients with 30 healthy individuals, and the difference in GLS results between the participants demonstrated that the FCN is sensitive to unwrapping left ventricular data in a heterogeneous cohort [30]. Moving forward with their work on GLS computation, Kar and her team investigated early alterations in prognostic factors such as GLS with standard Cox proportional hazards regression for estimating the risk of CTR-CVT incidents in patients with breast cancer undergoing cancer treatment using their previously developed AI-FCN.

Moving forward, Kar and her team carried out a trial using their tool to estimate the risk of developing cardiotoxicity in patients with breast cancer using data from their previous studies [31]. The trial proved their hypothesis that GLS computation can be used for early detection of CTR-CVT as an independent prognostic method of left ventricular dysfunction [31]. The advantage Kar et al had in their studies was that they were able to validate their solution internally within their center. However, their trials did not come without limitations. The solutions were not validated externally with other centers, and there was a greater sample for better accuracy measures [29-31]. In addition, the phase unwrapping approach for GLS measures was not compared to phase wrapping with another FCN, such as PhaseNet, which is considered a significant limitation in their conclusion [30].

Concurrently, in 2022, Chang et al [35] conducted another single-center prospective study and included a larger sample size of 211 patients diagnosed with breast cancer at different stages [35]. Chang et al [35] aimed to establish an AI-based predictive model for CTR-CVT using a cardio-oncology program. They prospectively collected clinical information and echocardiographic images from patients with breast cancer over 1 year. In their study, 2 echo technicians performed an echocardiogram independently to measure the LVEF at baseline, 3 months, 6 months, and 1 year after patients received their treatment. A cardiologist with a validated reliability and reproducibility interpreted the images. Moving forward with the AI solution, data were validated using a data mart for further analysis. Then, we compared the accuracy, precision, sensitivity, specificity, and area under the curve of the random forest, logistic regression, support vector clustering, LightGBM, K-nearest neighbour, and multilayer perceptron models. This process yielded the best accuracy in predicting CTR-CVT [35]. Moreover, the multilayer perceptron showed the best results in predicting heart failure with a reduced ejection fraction as an early sign of myocardial dysfunction after the occurrence of CTR-CVT [35].

Shen et al [34] conducted the most recent study in China in 2023. The study aimed to evaluate whether the pretreatment CACS can stratify the risk of CTR-CVT and MACEs in patients with diffuse large B-cell lymphoma (DLBCL). They retrospectively collected nongated and noncontrast chest CT scans of 1468 patients from 4 health centers in China, then used a deep-learning–based algorithm software (CACScoreDoc) to calculate the automatic CACS. CACScoreDoc automatically calculated the CACS and transmitted the results to the doctors after uploading the CT images to the software. The study showed that automating CACS derived from chest CT scans done before receiving the treatment is potentially helpful in identifying patients at risk of developing CTR-CVT and MACEs in patients with DLBCL receiving anthracycline chemotherapy, which can guide clinicians to implement cardiovascular protective strategies and minimize CTR-CVT in DLBCL patients [34].

Although cardiovascular events that are caused by cancer medications vary in prevalence from one type of cancer and its medication to another, they are still the second most common cause of mortality in cancer survivors. To accurately predict the risk of cardiotoxicity among individuals receiving cancer treatment is still a great challenge in the cardio-oncology field due to high cost, limited access to care, and inadequate compliance with screening protocols. Therefore, noninvasive, low-cost, accessible, innovative approaches to predict high-risk individuals and detect cardiotoxicity early among patients with cancer are critically needed to enable optimal screening, early diagnosis, and timely interventions [36].

### Current Versus Future AI Practice

The current tool used to investigate signs of cardiotoxicity is medical imaging, with the 2 most used imaging modalities for this purpose being the echocardiograph and CMR. However, although these modalities have helped the medical field to achieve significant improvement in prognosis in this area, some drawbacks hold them back from being optimal methods of investigation. The echocardiograph is entirely user-dependent in image reproducibility and results interpretation, leaving ample room for bias and inconsistency. On the other hand, the CMR is not always available due to its high cost. Therefore, more robust, cost-effective methods and imaging protocols are needed in this cardio-oncology area to optimize patient care [36].

Many health care disciplines have moved toward advancing artificial intelligence and developing better ML algorithms as they continue to improve patient care quality significantly. With the availability of enormous volumes of patient data and accessibility of proper hardware, AI and ML can accelerate the pace of change in health care. These technologies can sift through the data and analyze it much faster than humans, leading to increased efficiency. ML is used to predict clinical risk factors by feeding it with an enormous volume of data retrieved from patient medical records or national datasets and registries or detect cardiotoxicity via deep learning of patients’ cardiovascular images. In this review, the authors focused their assessment on using AI and ML in cardiovascular imaging to increase the diagnostic strength and accuracy in detecting CTR-CVT.

This review included 7 studies that intended to assess the implementation of AI in cardiovascular imaging among patients with cancer. These studies examine the use of AI on MRI, echocardiogram, and CT imaging modalities with different AI technologies such as ML, CNNs, and image segmentation.

The future of imaging AI in cardio-oncology holds substantial promise. This convergence of cutting-edge technologies, encompassing molecular imaging, wearable devices, multiomics data, and predictive modeling, is poised to transform cardiotoxicity management in patients with cancer. These advancements enable early detection and personalized risk assessment and promise targeted interventions, ultimately enhancing patient outcomes and survivorship. This future trajectory in imaging AI aligns with the significant advancements witnessed from ML to deep learning in AI, revolutionizing robotics and autonomous systems’ capabilities and enabling them to perceive, learn, and adapt with increased efficiency and accuracy in complex environments. These models, leveraging AI algorithms trained on diverse patient cohorts and multimodal imaging data, could assist clinicians in formulating proactive strategies for long-term cardiac care in cancer survivors, thereby enhancing overall cardiovascular health and quality of life.

### Challenges of AI in Health Care

As promising as AI and ML sound to the advancement of imaging in health care and the prediction of the risk of developing cardiotoxicity among patients receiving cancer treatment specifically, there are methodological and practical limitations preventing these technologies from reaching their full potential. The evidence base needs more prospective validation of the technology and current workflow, including evidence on the length of analysis required for validation and the interoperator and interobserver variability to eliminate manufactured variations that limit reproducibility [23]. Moreover, their usefulness in health care depends on incorporating the AI tool in clinical decision-making as part of the clinical practice routine, and that concern needs further investigation [37]. Another inadequacy of AI applications in health care is the systematic biases affecting patient demographics, such as gender imbalance [38]. It is worth mentioning that AI requires training on all kinds of populations with different demographics to guarantee equal performance from one population to another. It is recommended that multiple massive datasets be combined either retrospectively or prospectively to improve the generalizability of the ML process and the training of AI models, which was not achieved by all the included studies in this review [39].

### Review Limitations

The first limitation we had while conducting this review was the limited published evidence in the literature about the application of imaging AI in cardio-oncology to predict CTR-CVT. Therefore, we could not specify the cancer type or treatment under investigation. Second, even though there is significant literature on AI and imaging with different modalities, when we narrowed it down to our criteria, which was patients with cancer who are undergoing cardiotoxicity assessment, the literature search resulted in 3 different imaging modalities rather than studying AI with one specific imaging technique at a time. This resulted in different outcomes that prevented us from proceeding with a meta-analysis.

The use of AI in the medical field is a relatively new research area. This review could be used to stimulate further research. It can be used as groundwork for lab work to improve AI models or inspire new ones. In addition, this review highlights the positive outcomes of different studies in this area and their limitations. It may encourage experts to improve the AI and ML models and eventually implement them into medical imaging, possibly leading to the advancement of the field. However, given this field’s rapidly evolving nature, additional studies may have been published since the initial search process for this paper.

### Conclusions

In conclusion, this systematic review highlights the promising potential of AI in enhancing cardio-oncology imaging for predicting cardiotoxicity in patients with cancer. Through analyzing 7 studies conducted between 2018 and 2023, it became evident that AI methodologies, including ML and deep learning, can significantly improve the accuracy and efficiency of cardiotoxicity assessments across various imaging modalities, such as echocardiography and CMR.

The review underscores that AI-driven tools have demonstrated improved clinical outcomes by enabling earlier detection of cardiovascular complications associated with cancer therapies. However, while the findings are encouraging, the limited number of studies and their varying methodologies indicate a need for further research. This includes conducting larger, multicenter trials to validate AI applications in diverse patient populations and refine these technologies for routine clinical use.

In light of these insights, collaboration among data scientists, health care professionals, and researchers is essential to advancing AI’s integration in cardio-oncology. This collaboration will pave the way for personalized medicine approaches, ultimately enhancing patient care and improving the quality of life for cancer survivors at risk of cardiotoxicity.

## Supplementary material

10.2196/63964 Multimedia Appendix 1 Definitions.

10.2196/63964 Multimedia Appendix 2 Search strategy.

10.2196/63964 Checklist 1 PRISMA checklist.

## Abbreviations

AI: artificial intelligence
ASCO: American Society of Clinical Oncology
CACS: coronary artery calcium score
CLAIM: Checklist for Artificial Intelligence in Medical Imaging
CMR: cardiac magnetic resonance
CT: computed tomography
CTR-CVT: cancer therapy–related cardiovascular toxicity
CVD: cardiovascular disease
DCNN: deep convolutional neural network
DENSE: displacement encoding with stimulated echoes
DLBCL: diffuse large B-cell lymphoma
FCN: fully convolutional networks
GLS: global longitudinal strain
HER2: human epidermal growth factor receptor 2
LVEF: left ventricular ejection fraction
MACE: major adverse cardiovascular event
MeSH: Medical Subject Headings
ML: machine learning
MRI: magnetic resonance imaging
PRISMA: Preferred Reporting Items for Reviews and Meta-Analyses
PROSPERO: International Prospective Register of Systematic Reviews
ROBINS-I: risk of bias in nonrandomized studies - of interventions
