# Supplementary material for: Application of Artificial Intelligence in Cardio-Oncology Imaging for Cancer Therapy–Related Cardiovascular Toxicity: Systematic Review
Source: JMIR Cancer. 2025 May 9;11:e63964. doi: 10.2196/63964 (PMC12083731; doi:10.2196/63964)
Supplement: Multimedia Appendix 1 [file cancer-v11-e63964-s001.docx]

Appendix (A)

# Definitions

## Cancer treatment, modalities, side effects and prevention:

Cancer treatment aims to cure a type of cancer, shrink a tumour, or stop the progression of cancer [1]. It can be either cancer-directed or non-cancer-directed therapy; the decision depends on the kind and stage of cancer the patient was diagnosed with [2, 3]. The non-cancer-directed therapy is recommended to alleviate pain and relieve symptoms in cancer patients, but it does not treat cancer itself [2]. On the other hand, cancer-directed therapy is divided into three treatment approaches: 1) Curative treatment (also called primary), 2) Adjuvant treatment, and 3) Palliative treatment [2, 1]. The curative treatment is aimed to eradicate or kill the cancer completely; total hysterectomy is an example of this kind of treatment [2]. The adjuvant treatment is administered to complement and enhance the primary treatment to reduce the chance of its occurrence again [1]. Finally, palliative treatment is given to the patient to relieve symptoms caused by the cancer or any of its treatments [3].

Cancer-directed treatment has different modalities, including surgery, chemotherapy, radiotherapy, immunotherapy, hormone therapy, stem cell and bone marrow transplants, and targeted cancer drugs [2, 4]. These modalities can be done solely or as a combination of more than one.

### Cancer surgery:

Surgery is one of the main treatments for many types of cancer. Surgical intervention is done to establish a diagnosis, determine the extent of the disease, totally or partially remove a tumor or its secondary sites, and reduce the risk of future diagnosis [2, 5]. A surgical intervention does not come without risks such as bleeding, blood clots, damage to nearby tissues, drug reaction, pain, infection, damage to other organs, and slow recovery of other body functions [6]. To prevent these risks, surgeons are required to check the patient's lab tests for blood clotting normalcy and pay extra attention when working near blood vessels; patients are recommended to be mobile post-surgery to prevent blood clotting, intense monitoring of patients' heart rate and other signs from preventing a reaction to the anaesthesia or any other drug used during the surgery [6]. Moreover, the risk of incision site, lung infection – especially for patients with reduced lung function, or other infections can be prevented by good hygiene, deep breathing exercises, and antibiotics [6]. Other long-term effects may include jeopardizing the immune system when removing the spleen, phantom limb when amputating a limb, and lymphedema when removing lymph nodes [7].

### Chemotherapy:

It is one of the most common treatments for many cancer types. Chemotherapy uses a chemical or cytotoxic drug to kill fast-growing cancer cells to cure, control, or palliate cancer disease [2, 8]. Chemotherapy runs throughout the bloodstream; thus, it is considered a systemic treatment [8, 9]. Being a systemic drug, chemotherapy may also damage healthy cells, causing several unpleasant short-term side effects such as fatigue, anaemia, hair loss, skin irritation and dryness, nausea, vomiting, loss of appetite, cognitive and mental impairment, emotional distress, and many other effects [10, 11]. Some of these side effects cannot be prevented but can be managed. The clinician may decide to lower the dose administered, change the treatment, or halt the treatment altogether [12]. Other modalities of side effects management include medications, physical activity, cooling cap for hair loss, and dietary intake [12, 13]. However, many patients may develop long-term or late effects post-treatment, such as heart problems (cardiotoxicity), lung disease, dental problems, infertility, osteoporosis, intestinal problems, and nerve damage [7]. These side effects and the reasons they affect some patients and not others are not fully understood; thus, prevention and treatment strategies for long-term side effects of chemotherapy are not yet well-developed [14].

### Radiotherapy:

Radiotherapy, or radiation therapy, is another cancer treatment modality that utilizes high-energy radiation, specifically ionizing radiation such as X-rays, gamma rays, high-energy electrons, or heavy particles [15], to annihilate cancer cells [16]. In radiotherapy, the projected rays pass through the targeted tissues, thereby either directly killing cancer cells or inducing genetic alterations (DNA alteration), which inevitably cause the destruction of cancer cells and shrinking of tumours [17]. As radiation passes into the targeted tissue, both normal and cancer cells are exposed to radiation rays [18]. Both cell types could be harmed, thereby posing many risk factors such as mucosal ulcers, speech difficulties, headaches, radiation pneumonitis, infertility, and acute pericarditis if respective systems were irradiated. As radiation is a known carcinogen, long-term complications that have an annually increasing risk of 0.2% to 1% in cancer survivors are secondary malignancies [19]. Nowadays, Oncologists and radiotherapists intend to enhance radiotherapy techniques to decrease its risks rather than prevent radiotherapy's impact. These techniques include brachytherapy, fractionation, intraoperative radiation therapy, image-guided radiotherapy and intensity-modulated radiation therapy, stereotactic surgery and stereotactic body radiation therapy (SBRT), and targeted radionuclide therapy [18, 19]. Some of the techniques mentioned above are more often used for specific cancers, such as brachytherapy, which is composed of implanting radioactive pellets in which radiation is slowly released into a short distance to spare healthy tissue from the radiation exposure is used often with prostate and gynaecological malignancies [19]. Other techniques, such as SBRT, which avails itself of intensity-modulated radiation therapy and image-guided radiotherapy, project rays at precise and multiple angles to obliterate small-scale and clear-cut primary and metastatic tumours at any bodily region [20, 21].

### Immunotherapy:

Contrary to conventional cancer treatments like radiotherapy and chemotherapy, immunotherapy harnesses the ability of the body's immune system to prevent, control, and eliminate cancer [22]. Immune cells can significantly influence tumour surveillance and clearance [23]. Tumour-infiltrating lymphocytes are immune cells sometimes found in or around the cancerous tumour [24]. Although this is a sign that the body is responding and trying to slow or prevent cancer growth, it is not always developed in all cancer patients [24]. Moreover, cancer cells can mutate genetically for less visibility to the immune system, have proteins on their surface that can turn off immune cells, or change the normal cells around the tumour to interfere with the immune system's response to the tumour [24]. Well-known clinical applications of immunotherapy include immune checkpoint inhibitors (ICIs). Immune checkpoints are part of the body's immune system to prevent it from being too strong; ICI drugs block these checkpoints, allowing the immune cells to respond more strongly to the cancer cells [24]. Another immunotherapy type is T-cell transfer therapy, which boosts the body's natural T-cells to fight cancer. These monoclonal antibodies are artificial immune system proteins designed to bind to specific targets on cancer cells, allowing the immune system to detect and destroy them [25], chimeric antigen receptor T-cell therapy, cytokines, and tumour vaccines [26].

Due to its popularity, most evidence concerning the side effects of immunotherapy revolves around ICIs. The most common immune-related adverse effect of ICIs is dermatologic toxicity, which is reported to occur in 30%-50% of patients treated with ICIs [27]. Other common side effects include but are not limited to gastrointestinal toxicity (diarrhoea and colitis), hepatitis, endocrinopathies (thyroid toxicity, pituitary toxicity, adrenal insufficiency, and insulin-dependent diabetes mellitus), pneumonitis, and rheumatologic toxicity (arthritis, sicca syndrome, and myositis) [27]. It is important to note that the side effects vary depending on the specific ICI agent used. While the search for preventive measures in the case of immunotherapy is limited, there has been scarce research into periodic fast mimicking diet preventing the adverse effects of immunotherapy such as anaphylaxis; however, the study is more specific in 4T1 breast cancer, and the study is nevertheless in the animal trials [28].

### Hormone therapy (Endocrine therapy):

Hormones are proteins or natural substances made by glands in the body that help to control the growth and activity of specific cells and organs [29]. Hormone therapy is a modality of cancer treatment that takes advantage of the interplay of hormones in specific hormone-dependent cancers, including breast, prostate, ovarian and uterine cancer [30]. Hormone therapy is considered another systemic treatment because of its circulation throughout the body, blocking or reducing the amount of hormones to stop or slow down cancerous growth [31]. The type of hormone therapy used depends on the type of cancer and how advanced the disease is. Hormones can be given orally, by injection, or surgically to remove the hormone-producing organ, and it will either block the body's ability to produce hormones completely or interfere with the hormone's behaviour in the body [32]. This kind of treatment is the standard choice for non-life-threatening advanced-stage cancer, estrogen-receptor-positive cancers, or patients who are not fit for other aggressive treatment modalities such as chemotherapy [33].

Although hormone therapy is considered the least aggressive choice of treatment with low morbidity [34], it has a share of side effects, depending on the type of cancer and the type and dose of hormone the patient is receiving [31]. Common side effects for men (prostate cancer treatment) and women (breast or endometrial cancer treatment) are hot flashes, decreased sexual drive, fatigue, bone loss and higher risk of fractures [31]. In addition, men may have memory problems, erectile dysfunction, weight gain, and increased risk of other health problems [32], while women might have vaginal dryness or irritation, nausea, pain in muscles and joints, and a higher risk of developing stroke, blood clots, cataracts, and heart disease [31]. Nonetheless, some protocols can mitigate or prevent these side effects. For instance, clinicians might consider altering from one aromatase inhibitor to another to decrease musculoskeletal side effects [30]. Moreover, some patients may consider pharmacological approaches for their alleviated vasomotor, such as gabapentin and antidepressants (Serotonin and norepinephrine reuptake inhibitors and Selective serotonin reuptake inhibitors). In contrast, other patients may consider non-pharmacological solutions such as physical activity and weight loss [35].

### Stem cell transplant (SCT):

Also called "Bone marrow transplant"; is a procedure where the patient receives healthy stem cells to replace the diseased stem cells after conditioning treatment [36]. Stem cells are immature cells created in the bone marrow to form any blood cell (white and red blood cells and platelets) the body needs [36, 37]. Conditioning treatment is a high dose of chemotherapy, occasionally combined with radiotherapy received by the patient to destroy any remaining cancer cells in the body, suppress the patient's immune system to reduce graft rejection, and allow room for the transplanted stem cells in the bone marrow [38]. SCT treats patients with haematological cancers, such as leukaemia, lymphoma and myeloma [39]. The main side effects of SCT are caused by chemotherapy and radiotherapy, which are discussed previously. Patients receiving SCT are at an increased risk of infection due to the lack of white blood cells after the conditioning treatment, which can be treated with antibiotics [40].

Moreover, when receiving an allogeneic transplant (from a donor), patients might develop a severe adverse effect called graft-versus-host disease, where white blood cells from the graft recognize cells in the host body as foreign bodies and attack them [37]. This disease can cause significant damage to the organs a few weeks after the transplant or much later. Graft-versus-host disease can be managed by steroids or any other immune suppressant drugs [37].

### Targeted therapy:

Unlike chemotherapy, which affects both malignant and normal cells, targeted therapy focuses on malignant cells, hence the name. This type of therapy targets specific traits only available in the targeted cells, providing more tailored treatment [41]. Targeted therapies fall into two major categories. The first comprises synthetic antibodies called "monoclonal antibodies", which bind to particular proteins such as receptors and inhibit their function [42]. The second category consists of small molecular inhibitors that enable them to enter cells, explicitly targeting proteins essential for cellular homeostasis, such as tyrosine kinase inhibitors [42]. Trastuzumab is an example of targeted therapy, a monoclonal antibody that targets HER2 receptors, expressing breast cancer cells [43]. The fact that 25-30% of breast cancer cases express HER2 receptors emphasizes the importance of tailored therapy [44]. Targeted therapy is more precise and tailored, but there are significant disadvantages and factors to consider. Firstly, Trastuzumab is known to potentially cause CTR-CVT [45]. Secondly, as each case differs, not every patient may benefit from targeted therapy. Therefore, before starting targeted therapy, a thorough prognostic evaluation should be completed [42].

## Cancer therapy-related cardiovascular toxicity, risk factors, management, and prevention:

There is no definite definition for cancer therapy-related cardiovascular toxicity (CTR-CVT); however, various organizations have defined it differently based on threshold changes in LVEF. For instance, The National Cancer Institution defines it as "toxicity that affects the heart". The American Society of Echocardiography, as well as the European Association of Cardiovascular Imaging, define cardiotoxicity as a decline of 10% to 53% in LVEF [46], while The American College of Cardiology defines cardiotoxicity as "a serial decline in LVEF" [47].

Cancer therapy-related cardiovascular toxicity can be symptomatic or asymptomatic, and the spectrum varies from mild heart failure symptoms with no intensification of therapy required to very severe heart failure with transplantation consideration [48]. It is difficult to determine the true incidence of CTR-CVT due to the variation in cancer treatment regimen, doses, and data sources (clinical trials or cohort studies) [47]. Thus, it is crucial to identify high-risk patients prior to cancer therapy to anticipate, prepare, prevent, and manage cardiotoxicity events. The risk factors associated with CTR-CVT are based on the patient's lifestyle, demographics, medical history, and type of therapy.

The Journal of American Heart Association, through a pragmatic approach to the American and European Cardio-Oncology guidelines, categorized the risk factors into modifiable and Non-modifiable risks. Non-modifiable risks include patients with previous cardiovascular disease, aged over sixty, and elevated cardiac biomarkers before initiating cancer treatment (N‐terminal pro‐B‐type natriuretic peptide (or B‐type natriuretic peptide) and/or troponin). On the other hand, modifiable risks include smoking, hypertension, diabetes mellitus, obesity, dyslipidemia, and chronic renal insufficiency [49, 50]. Moreover, severe treatment plans can increase the risks of CTR-CVT, such as patients receiving high-dose anthracycline, high-dose radiotherapy, or lower-dose anthracycline, HERis, VEGFis, proteasomes, or Bcr‐Ablis with least two of the pre-existing characteristics risk factors [49].

Advanced imaging modalities such as Echocardiography and cardiac magnetic resonance imaging (CMRI) can assess cardiac strain. These imaging modalities can detect myocardial strain at a subclinical level, allowing the medical team to form an intervention or adjustment if needed [51, 52]. Suppose a patient were to suffer from CTR-CVT. In that case, management plans include: 1) adjusting the cancer treatment, whether that is by changing the drug into an alternative or by adjusting the dose to a level where it causes less cardiac strain [53], 2) dealing with modifiable risk factors such as smoking and obesity [53], 3) Prescribing cardioprotective medications such as Angiotensin-converting enzyme inhibitors and beta-blockers where appropriate [53], 4) Assembling a multidisciplinary team, a fundamental step in dealing with CTR-CVT. The collaboration between the cardiology and oncology departments is vital for adjusting all the previous mentions and developing a more personalized treatment plan for the patient, and 5) using advanced imaging techniques to surveillance and manage CTR-CVT [54]. Table A illustrates the imaging techniques used for the risk assessment before and during treatment.

Table A: Summary table of imaging modalities, their definition, and utility

| **Imaging modalities** | **Definition** | **Risk assessment utility** | **Treatment utility** |
| --- | --- | --- | --- |
| **Echocardiography** | It is an accessible real-time imaging modality used to assess structural integrity and function of the heart chambers by ultrasound, making it a non-invasive modality that does not use radiation. It is the most used modality for assessing CTIC [51, 54]. | Measures the LVEF and GLS to assess for ischemia, LV/RV function, and give a 2D/3D baseline strain assessment [54]. | - Monitor subclinical cardiotoxicity strain. - Surveillance for CTR-CVT [51, 54]. |
| **Cardiac magnetic resonance imaging (CMR)** | It is a non-invasive modality that uses a powerful magnet and radio waves to image the heart's anatomy and function. It is used when there is high suspicion of cardiotoxicity in the patient [51]. | CMR uses many techniques, such as tissue characterization and measurement of cardiac function, to assess the risk of cardiotoxicity and myocardial strain [54]. | - Monitor suspicion of valvular dysfunction, LV/RV dysfunction. - Surveillance of CTIC [51, 54]. |
| **Multigated acquisition (MUGA)** | A nuclear cardiology technique tracks the motion of the radiolabelled cells that picked up the radioactive tracer using a gamma camera. The imaging generated by MUGA can allow us to assess LVEF, which can be interpreted for cardiotoxicity. However, MUGA is not used to diagnose CTR-CVT [51]. | MUGA can measure LV function and micro- or microvascular anaemia [54]. | - Used for surveillance of CTR-CVT by assessing the LVEF [51, 54]. |
| **Cardiac computed tomography (CCT)** | A non-invasive modality that uses X-rays to create cross-sectional images of the heart. Allowing the medical team to assess for cardiovascular pathologies [51]. | It is not typically used for risk assessment of cardiotoxicity, unlike CMR and ECG [54]. | Used if there is high suspicion of coronary artery disease or pericardial disease [51, 54]. |

## Artificial intelligence (AI) in healthcare and its implications in cardio-oncology imaging

Artificial intelligence in healthcare may improve diagnosis accuracy and personalized, targeted treatment development. The implication of artificial intelligence in medical imaging is gaining attraction [55]. Hence, its significance to cardio-oncology. AI not only uses LVEF and GLS as parameters to predict and measure cardiotoxicity GLS but also can sift through and analyze data much faster than humans can, leading to increased efficiency of early diagnoses and treatment. Baseline LVEF and GLS assessment can detect LV contractile function changes in patients undergoing CTR-CVT. With advancements in computing infrastructures and AI algorithms, machine learning and digitalized data acquisition, the accurate procurement of these parameters is possible and desirable [51].

Applying AI and machine learning to imaging in cardio-oncology would utterly transform the field. The intelligent algorithms can efficiently process large amounts of image databases, allowing them to be familiar and point to pathologies/abnormalities [51]. Which, in return, can lead to improved accuracy in data acquisition, Optimized bedside imaging, and Improved prediction and early detection of cardiac dysfunction [52]. Deep learning models and AI are generally revolutionizing medical imaging and, if availed correctly, will improve patient care and clinical practice by exploring venues that exclusively rely on human expertise, which indicates that the opportunities are endless in the cardio-oncology field and medicine in general [51].

# References

1. Mayo Clinic. Cancer treatment. https://www.mayoclinic.org/tests-procedures/cancer-treatment/about/pac-20393344. Published May 25, 2022. Accessed July 19, 2023.
2. Esteban D, Whelan S, Laudico A, Parkin DM. Chapter 2 – The diagnosis and treatment of cancer. In: Manual for Cancer Registry Personnel: IARC Technical Report No. 10. Lyon: International Agency for Research on Cancer; 1995.
3. Centers for Disease Control and Prevention (CDC). Cancer treatments. https://www.cdc.gov/cancer/survivors/patients/treatments.htm. Published May 15, 2023. Accessed July 19, 2023.
4. Cancer Research UK. Treatment for cancer. https://www.cancerresearchuk.org/about-cancer/treatment. Published July 5, 2021. Accessed July 19, 2023.
5. Cancer Research UK. What is cancer surgery? https://www.cancerresearchuk.org/about-cancer/treatment/surgery/about. Published May 18, 2022. Accessed July 19, 2023.
6. American Cancer Society (ACS). Risks of cancer surgery. https://www.cancer.org/cancer/managing-cancer/treatment-types/surgery/risks-of-cancer-surgery.html. Published October 2, 2019. Accessed July 23, 2023.
7. American Society of Clinical Oncology (ASCO). Long-term side effects of cancer treatment. https://www.cancer.net/survivorship/long-term-side-effects-cancer-treatment. Published September 2019. Accessed July 24, 2023.
8. American Cancer Society (ACS). How is chemotherapy used to treat cancer? https://www.cancer.org/cancer/managing-cancer/treatment-types/chemotherapy/how-is-chemotherapy-used-to-treat-cancer.html. Published November 22, 2019. Accessed July 19, 2023.
9. Cancer Research UK. What is chemotherapy? https://www.cancerresearchuk.org/about-cancer/treatment/chemotherapy/what-chemotherapy-is. Published June 10, 2020. Accessed July 19, 2023.
10. American Cancer Society (ACS). Managing cancer-related side effects. https://www.cancer.org/cancer/managing-cancer/side-effects.html. Accessed July 24, 2023.
11. National Health Service (NHS). Side effects - chemotherapy. https://www.nhs.uk/conditions/chemotherapy/side-effects/. Published May 25, 2023. Accessed July 24, 2023.
12. Yale Medicine. Side effects of cancer treatment. https://www.yalemedicine.org/conditions/side-effects-cancer-treatment. Accessed July 24, 2023.
13. Centers for Disease Control and Prevention (CDC). Side effects of cancer treatment. https://www.cdc.gov/cancer/survivors/patients/side-effects-of-treatment.htm. Published May 15, 2023. Accessed July 24, 2023.
14. Nurgali K, Jagoe RT, Abalo R. Editorial: Adverse effects of cancer chemotherapy: Anything new to improve tolerance and reduce sequelae? Front Pharmacol. 2018;9:245.
15. International Atomic Energy Agency (IAEA). What is radiation therapy? https://www.iaea.org/newscenter/news/what-is-radiation-therapy. Published April 18, 2023. Accessed July 30, 2023.
16. National Cancer Institute (NCI). Radiation therapy to treat cancer. https://www.cancer.gov/about-cancer/treatment/types/radiation-therapy. Published January 8, 2019. Accessed July 30, 2023.
17. Cancer Research UK. What is radiotherapy? https://www.cancerresearchuk.org/about-cancer/treatment/radiotherapy/what-is-radiotherapy. Published November 6, 2020. Accessed July 30, 2023.
18. Baskar R, Lee KA, Yeo R, Yeoh K. Cancer and radiation therapy: Current advances and future directions. Int J Med Sci. 2012;9(3):193-199.
19. Majeed H, Gupta V. Adverse effects of radiation therapy. In: StatPearls [Internet]. Treasure Island (FL): StatPearls Publishing; 2023.
20. Lo SS, Fakiris AJ, Chang EL, et al. Stereotactic body radiation therapy: A novel treatment modality. Nat Rev Clin Oncol. 2010;7(1):44-54.
21. Tipton K, Launders JH, Inamdar R, Miyomoto C, Schoelles K. Stereotactic body radiation therapy: Scope of the literature. Ann Intern Med. 2011;154(11):737-745.
22. Cancer Research Institute (CRI). Immunotherapy: Impacting all cancers. https://www.cancerresearch.org/immunotherapy-by-cancer-type. Accessed July 30, 2023.
23. Tan S, Li D, Zhu X. Cancer immunotherapy: Pros, cons and beyond. Biomed Pharmacother. 2020;124:109821.
24. National Cancer Institute (NCI). Immunotherapy to treat cancer. https://www.cancer.gov/about-cancer/treatment/types/immunotherapy. Published September 24, 2019. Accessed July 30, 2023.
25. Abbott M, Ustoyev Y. Cancer and the immune system: The history and background of immunotherapy. Semin Oncol Nurs. 2019;35(5):150923.
26. Cancer Research UK. Types of cancer immunotherapy. https://about-cancer.cancerresearchuk.org/about-cancer/treatment/immunotherapy/types. Published January 20, 2021. Accessed July 30, 2023.
27. Kennedy LB, Salama AKS. A review of cancer immunotherapy toxicity. CA Cancer J Clin. 2020;70(2):86-104.
28. Longo V. Periodic fasting mimicking diet, longevity, and disease. Innov Aging. 2022;6(Suppl 1):91.
29. Cancer Research UK. Hormone therapy for cancer. https://www.cancerresearchuk.org/about-cancer/treatment/hormone-therapy/for-cancer. Published January 27, 2021. Accessed July 30, 2023.
30. Ulm M, Ramesh A, McNamara KM, et al. Therapeutic advances in hormone-dependent cancers: Focus on prostate, breast and ovarian cancers. Endocr Connect. 2019;8(2):R10-R26.
31. American Cancer Society (ACS). Hormone therapy. https://www.cancer.org/cancer/managing-cancer/treatment-types/hormone-therapy.html. Published July 21, 2020. Accessed July 30, 2023.
32. National Cancer Institute (NCI). Hormone therapy to treat cancer. https://www.cancer.gov/about-cancer/treatment/types/hormone-therapy. Published August 23, 2022. Accessed July 30, 2023.
33. Lumachi F, Luisetto G, Basso SM, et al. Endocrine therapy of breast cancer. Curr Med Chem. 2011;18(4):513-522.
34. Larionov AA, Miller WR. Challenges in defining predictive markers for response to endocrine therapy in breast cancer. Future Oncol. 2009;5:1415-1428.
35. Condorelli R, Vaz-Luis I. Managing side effects in adjuvant endocrine therapy for breast cancer. Expert Rev Anticancer Ther. 2018;18(11):1101-1112.
36. Leukemia & Lymphoma Society (LLS). Stem cell transplantation. https://www.lls.org/treatment/types-treatment/stem-cell-transplantation. Accessed August 1, 2023.
37. National Cancer Institute (NCI). Stem cell transplants in cancer treatment. https://www.cancer.gov/about-cancer/treatment/types/stem-cell-transplant. Published April 19, 2015. Accessed August 1, 2023.
38. American Cancer Society (ACS). Getting a stem cell or bone marrow transplant. https://www.cancer.org/cancer/managing-cancer/treatment-types/stem-cell-transplant/process.html#:~:text=Conditioning%20treatment%20(chemo%20and%2For%20radiation%20therapy),-Conditioning%2C%20also%20known&text=It%27s%20done%20for%20one%20or,cells%20in%20th. Published March 20, 2020. Accessed August 1, 2023.
39. Dessie G, Molla MD, Shibabaw T, Ayelign B. Role of stem-cell transplantation in leukemia treatment. Stem Cells Cloning. 2020;13:67-77.
40. American Cancer Society (ACS). Stem cell or bone marrow transplant side effects. https://www.cancer.org/cancer/managing-cancer/treatment-types/stem-cell-transplant/transplant-side-effects.html. Published March 20, 2020. Accessed August 1, 2023.
41. Baskar R, Lee KA, Yeo R, Yeoh K. Targeted therapy for cancer. J Cancer Mol. 2006;2(2):57-66.
42. Tsimberidou AM. Targeted therapy in cancer. Cancer Chemother Pharmacol. 2015;76:1113-1132.
43. Mohamed A, Krajewski K, Cakar B, Ma CX. Targeted therapy for breast cancer. Am J Pathol. 2013;183(4):1096-1112.
44. National Cancer Institute (NCI). HER2 genetic link to breast cancer. https://www.cancer.gov/research/progress/discovery/her. Published April 11, 2018. Accessed December 11, 2023.
45. Gernaat SAM, Ho PJ, Rijnberg N, et al. Risk of death from cardiovascular disease following breast cancer: A systematic review. Breast Cancer Res Treat. 2017;164(3):537-555.
46. Seidman AH, Hudis CA, Pierri MK, et al. Cardiac dysfunction in the trastuzumab clinical trials experience. J Clin Oncol. 2002;20(5):1215-1221.
47. Lambert J, Thavendiranathan P. Controversies in the definition of cardiotoxicity: Do we care? Am Coll Cardiol. 2016;7/7/2016.
48. Task force on cardio-oncology of the European Society of Cardiology. 2022 ESC guidelines on cardio-oncology developed in collaboration with the European Hematology Association (EHA), the European Society for Therapeutic Radiology and Oncology (ESTRO) and the International Cardio-Oncology Society (IC-OS). Eur Heart J. 2022;43:422-4361.
49. Alexandre J, Cautela J, Ederhy S, et al. Cardiovascular toxicity related to cancer treatment: A pragmatic approach to the American and European cardio-oncology guidelines. J Am Heart Assoc. 2020;9(18):e018403.
50. Neilan TG, Asnani A, Tripathy D, Scherrer-Crosbie M. Risk and prevention of anthracycline cardiotoxicity. UpToDate. June 15, 2023. https://www.uptodate.com/contents/risk-and-prevention-of-anthracycline-cardiotoxicity. Accessed July 24, 2023.
51. Madan N, Lucas J, Akhter N, et al. Artificial intelligence and imaging: Opportunities in cardio-oncology. Am Heart J Plus. 2022;15:100126.
52. Yagi R, Goto S, MacRae CA, Deo RC. Expanded adaptation of an artificial intelligence model for predicting chemotherapy-induced cardiotoxicity using baseline electrocardiograms. Eur Heart J. 2022;43(Supplement_2):ehac544.2577.
53. Grieve DJ, Davidson SM. New insights into cardiotoxicity caused by chemotherapeutic agents. Br J Pharmacol. 2017;174(21):3675-3676.
54. Baldassarre LA, Ganatra S, Lopez-Mattei J, et al. Advances in multimodality imaging in cardio-oncology: JACC state-of-the-art review. J Am Coll Cardiol. 2022;80(16):1560-1578.
55. Manaswi N. Medical imaging being transformed with GAN: MRI to CT scan and many others. https://medium.com/analytics-vidhya/medical-imaging-being-transformed-with-gan-mri-to-ct-scan-and-many-others-18a307ef528. Published March 18, 2020. Accessed September 17, 2023.
