# Supplementary material for: Application of Artificial Intelligence in Cardio-Oncology Imaging for Cancer Therapy–Related Cardiovascular Toxicity: Systematic Review
Source: JMIR Cancer. 2025 May 9;11:e63964. doi: 10.2196/63964 (PMC12083731; doi:10.2196/63964)
Supplement: Multimedia Appendix 2 [file cancer-v11-e63964-s002.docx]

Appendix (B)

# Search Strategy

## Concepts

1. ((Artificial Intelligence) OR (AI) AND (Cardio-Oncology))
2. ((Artificial Intelligence) OR (AI)) AND ((Cardiotoxicity) OR (Cardiac toxicity)) AND (Anthracycline) AND (Cancer)
3. ((Artificial Intelligence) OR (AI)) AND ((Cardiotoxicity) OR (Cardiac toxicity)) AND (Anthracycline)
4. ((Artificial Intelligence) OR (AI)) AND ((Cardiotoxicity) OR (Cardiac toxicity)) AND (Chemotherapy)
5. (Artificial Intelligence) OR (AI) AND (Cardio-Oncology) AND (Echo*)

## Search process

The first, second, and third authors independently searched the databases based on the agreed-upon concepts. Each author created their own database independently. HM used MS Excel, applying each search concept to all accessible databases outlined in the search strategy. The results from each search were exported from the databases and imported into separate sheets within the Excel workbook. After the search, all articles were consolidated into one "final" sheet, resulting in 617 journal articles after removing duplicates. MR and AR used EndNote 20 to identify and discard duplicates automatically. Furthermore, HM and MR manually verified the removed records to ensure accuracy.

The senior author then joined this stage to assist in reviewing the titles and abstracts. Any disagreements regarding study inclusion were discussed and resolved among the four authors.

The first and senior authors have extensive experience conducting systematic literature reviews, with several published works. Additionally, the authors consulted with experts through Editage services to ensure high reliability.

### Search Terms and Keywords:

We used free-text terms and Medical Subject Headings (MeSH) where applicable. The keywords were selected to comprehensively cover the topic of artificial intelligence (AI) in cardio-oncology imaging for predicting cardiotoxicity. The complete list of search terms is included in section 1.3 of Appendix B.

### Filters and Limits:

1. The search was limited to studies in the English language.
2. We included only original research articles (excluding case reports, reviews, grey material, and editorials).
3. The date range was from inception to June 2023.

### Use of Quotations:

For databases that support exact phrase matching (such as PubMed and Ovid Medline), quotation marks were used around multi-word search terms (e.g., "artificial intelligence", "cardio-oncology", "machine learning").

### Database-Specific Search Strategy:

Each database (PubMed, Ovid Medline, Cochrane Library, CINAHL, Google Scholar) had slightly modified search strings to align with their specific syntax and search engine functionalities.

## Search terms

| **Database** | **Terms** | **Fields** | **Results** |
| --- | --- | --- | --- |
| PubMed | artificial intelligence, AI, deep learning, machine learning, machine intelligence, computational intelligence, Convolutional neural networks, CNN, computer vision system*, algorithm*, cardio-oncolog*, cardiooncolog*, cardiovascular oncolog*, cardiotoxicit*, cardiac toxicit*, cardiovascular toxicit*, cancer treatment, cancer therap*, anthracycline*, chemotherapy, cytotoxic regimens, immunotherapy, Cancer treatment, therapy-induced, Echocardiogram, Echo*, imag*, MRI, magnetic resonance, cardiac magnetic resonance imaging, CMR, multigated acquisition, MUGA, Cardiac computed tomography, CCT | All | 593 |
| EBSCO/ CINAHL Ultimate | Artificial intelligence, AI, AI, machine learning, Algorithms, Cardiotoxicity, cardio-toxicity, cardiovascular toxicity, cancer, cardio-oncology, anthracycline*, imag*, MRI, magnetic resonance, echo*, cytotoxic regimens, immunotherapy, Cancer treatment, therapy-induced, Echocardiogram, Echo* | All | 2 |
| OVID Medline | AI, Artificial, Intelligence, Cardio-oncology, anthracycline*, imag*, MRI, magnetic resonance, echo*, cytotoxic regimens, immunotherapy, Cancer treatment, therapy-induced, Echocardiogram, MRI, magnetic resonance, echo, chemotherapy, cardiotoxicity, cardiac toxicity, anthracycline, | Keywords and Title | 267 |
| Cochrane Library  Included: Embase,  PubMed,  ClinicalTrial.gov, | Algorithms, Adaptive Algorithms, Artificial Intelligence, AI, Intelligent Systems, Machine Learning, Natural Language Processing, Neural Networks, Pattern Analysis, Prediction Methods, Classification Algorithms, Clustering Algorithms  Detection Algorithms, Multiple-Instance Learning Algorithms, Prediction Algorithms, Segmentation, Cardiac-Gated Imaging Techniques, Cardiac-Gated Single-Photon Emission Computer-Assisted Tomography, Gated Blood-Pool Imaging, Echocardiography, cardiotoxicity, cardiotoxicities, cardio-oncology, anthracycline, chemotherapy, immunotherapy | All Text | 16 |
| Other:  Google Scholar,  Elicit | AI, Artificial, Intelligence, Cardio-oncology, anthracycline*, imag*, MRI, magnetic resonance, echo*, cytotoxic regimens, immunotherapy, Cancer treatment, therapy-induced, Echocardiogram, MRI, magnetic resonance, echo, chemotherapy, cardiotoxicity, cardiac toxicity, anthracycline, | All | 21 |
